# Differential effects of natural rewards and pain on vesicular glutamate transporter expression in the nucleus accumbens

**DOI:** 10.1186/1756-6606-6-32

**Published:** 2013-07-09

**Authors:** David S Tukey, Michelle Lee, Duo Xu, Sarah E Eberle, Yossef Goffer, Toby R Manders, Edward B Ziff, Jing Wang

**Affiliations:** 1 Department of Biochemistry, New York University School of Medicine, New York, New York, USA; 2 Department of Anesthesiology, New York University School of Medicine, New York, New York, USA; 3 Current address: Columbia University College of Physicians and Surgeons, New York, New York, USA

**Keywords:** Vesicular glutamate transporters (VGLUTs), Nucleus accumbens, Glutamate, Natural rewards, Pain

## Abstract

**Background:**

Pain and natural rewards such as food elicit different behavioral effects. Both pain and rewards, however, have been shown to alter synaptic activities in the nucleus accumbens (NAc), a key component of the brain reward system. Mechanisms by which external stimuli regulate plasticity at NAc synapses are largely unexplored. Medium spiny neurons (MSNs) from the NAc receive excitatory glutamatergic inputs and modulatory dopaminergic and cholinergic inputs from a variety of cortical and subcortical structures. Glutamate inputs to the NAc arise primarily from prefrontal cortex, thalamus, amygdala, and hippocampus, and different glutamate projections provide distinct synaptic and ultimately behavioral functions. The family of vesicular glutamate transporters (VGLUTs 1–3) plays a key role in the uploading of glutamate into synaptic vesicles. VGLUT1-3 isoforms have distinct expression patterns in the brain, but the effects of external stimuli on their expression patterns have not been studied.

**Results:**

In this study, we use a sucrose self-administration paradigm for natural rewards, and spared nerve injury (SNI) model for chronic pain. We examine the levels of VGLUTs (1–3) in synaptoneurosomes of the NAc in these two behavioral models. We find that chronic pain leads to a decrease of VGLUT1, likely reflecting decreased projections from the cortex. Pain also decreases VGLUT3 levels, likely representing a decrease in projections from GABAergic, serotonergic, and/or cholinergic interneurons. In contrast, chronic consumption of sucrose increases VGLUT3 in the NAc, possibly reflecting an increase from these interneuron projections.

**Conclusion:**

Our study shows that natural rewards and pain have distinct effects on the VGLUT expression pattern in the NAc, indicating that glutamate inputs to the NAc are differentially modulated by rewards and pain.

## Background

Natural rewards and pain are represented in the brain with opposite hedonic values. Chronic administration of rewards leads to seeking and craving [1]. Pain, on the other hand, triggers avoidance behavior, and chronic pain can even lead to depression and anxiety [2]. The molecular basis for the behavioral changes observed after chronic presentation of natural reward and pain, however, remains incompletely understood. The nucleus accumbens (NAc) is a key component of brain reward circuitry [1,3]. Recent works from our and other labs show that medium spiny neurons (MSNs) in the NAc undergo synaptic modifications in the presence of natural rewards or drugs of addiction [4-7], and that synaptic plasticity in the NAc modulates craving for natural rewards or drugs of abuse [6,8]. We have recently shown, in particular, that in the presence of a natural reward such as sucrose, glutamate signaling in the NAc is augmented through synaptic incorporation of calcium permeable AMPA receptors (CPARs) [7]. At the same time, the NAc has also been shown by human imaging and animal studies to be activated by pain [9,10]. Furthermore, there is evidence that activation of the NAc in pain states is involved in avoidance behaviors. Thus, the NAc is an important region for both reward- and pain-induced behavioral plasticity.

The NAc receives afferent inputs from multiple cortical and subcortical structures, including the prefrontal cortex (PFC), thalamus, hippocampus, amygdala, and ventral tegmental area (VTA) [3]. The PFC, thalamus, hippocampus, and amygdala provide key glutamate inputs to the NAc, whereas the VTA is the main source for dopamine. A complex interplay of glutamate and dopamine inputs modulates the synaptic functions of the NAc [11-15]. There is emerging evidence that appetitive and aversive stimuli provide distinct dopaminergic inputs to the NAc [16,17]. However, the glutamatergic inputs to the NAc have not been well characterized in the presence of natural rewards or pain.

The accumulation of presynaptic glutamate in secretory vesicles is mediated by vesicular glutamate transporters (VGLUTs). VGLUTs (1–3) are a family of glutamate transport molecules, and these transporters control glutamate release from presynaptic neurons [18,19]. The levels of VGLUT expression have been correlated with levels of glutamate at the synaptic cleft [18]. Members of the VGLUT family also have distinct expression patterns. VGLUT1 and VGLUT2 are expressed in glutamatergic neurons in a complementary manner [20,21]. In the brain, VGLUT1 is primarily found in the cerebral cortex, hippocampus, and cerebellar cortex nuclei [21-25]. In contrast, VGLUT2 is mainly expressed in the thalamus, brainstem and deep cerebellar nuclei [21-23,25]. In addition, VGLUT2 has been found in dopaminergic neurons, in particular, in dopaminergic neurons from the VTA [26-29]. A third member of the VGLUT family, VGLUT3, is found in serotonergic neurons in the raphe nuclei and a subset of GABAergic interneurons in the cortex and hippocampus [20,30,31]. This primarily non-glutamatergic distribution of VGLUT3 suggests that it may in fact be a co-transporter of glutamate and other inhibitory or modulatory molecular signals such as GABA and serotonin [32]. In particular, VGLUT3 is also expressed on cholinergic interneurons in the NAc [33,34]. These cholinergic interneurons have been shown to play a critical role for the function of the NAc [35-37], and VGLUT3 likely regulates the co-release of acetylcholine and glutamate to influence the synaptic function in these neurons [33,34].

Because of the distinct expression pattern of the VGLUTs 1–3, these proteins can serve as presynaptic markers to understand glutamate inputs to the NAc under conditions of rewards or pain. We have developed a sucrose self-administration paradigm in rats to model chronic natural rewards, and we have recently shown that this paradigm induces psychomotor hyperactivity, a behavioral phenotype that is indicative of reward seeking [7]. In addition, we found that this paradigm results in the formation of CPARs, an important form of synaptic modification in the NAc. Transmission through these newly formed receptors, in turn, regulates reward seeking behaviors [7]. At the same time, we have also developed spared nerve injury (SNI) to model chronic pain in rats, and we have shown that this model leads to depression-like behaviors in addition to sensory allodynia [38]. In the current study, we examine VGLUTs levels in the NAc using these two rat models. We find that chronic pain decreases VGLUT1 and 3 levels, whereas repeated sucrose intake causes an increase in VGLUT3. These data suggest that pain causes a decrease in cortical inputs to the NAc. Meanwhile, co-release of glutamate with GABA, serotonin, and/or acetylcholine at NAc synapses is potentially decreased by chronic pain, but increased by natural rewards.

## Results

### Chronic pain decreases VGLUT1 and VGLUT3 expression in the NAc

**Figure 1:**
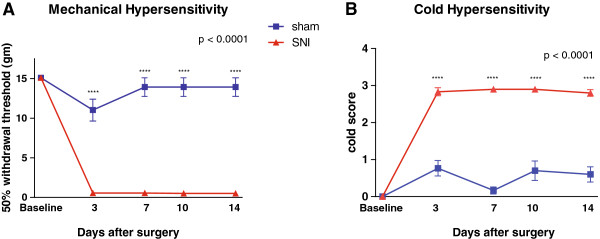
**SNI Causes Mechanical and Cold Hypersensitivity After Surgery. (A)** SNI-operated rats developed mechanical hypersensitivity after surgery. Two-way ANOVA with repeated measures, followed by Bonferroni post-test, n = 6, p < 0.0001. **(B)** Rats developed cold hypersensitivity after SNI. n = 6, p < 0.0001. Error bars show mean and s.e.m.

We used the SNI model, a peripheral nerve injury model for chronic neuropathic pain in adult male rats [38]. In this model, we surgically resected common peroneal and tibial branches of the sciatic nerve, leaving the sural nerve intact. 3 days after SNI, rats experienced abnormal hypersensitivity to mechanical and cold stimuli (Figure 1A and 1B), classic sensory indices for pain. Sensory hypersensitivity after SNI was chronic, persisting for at least 14 days (p < 0.0001) [38]. In contrast, control rats which underwent sham operation did not show this sensory hypersensitivity.

**Figure 2:**
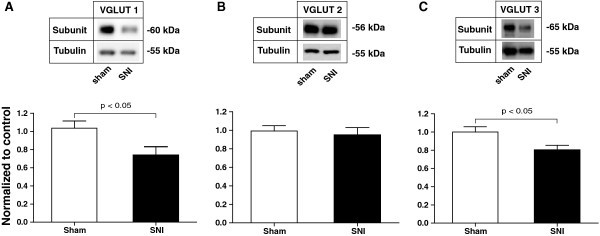
**Chronic Pain Causes a Selective Decrease in VGLUT1 and VGLUT3 Expression in the NAc. (A)** SNI caused a decrease in VGLUT1 subunits in the synaptoneurosomes of NAc. Student’s t test, n=15, p < 0.05. **(B)** SNI caused no changes in VGLUT2 subunits in the synaptoneurosomes of the NAc. n = 15, p > 0.05. **(C)** SNI caused a decrease in VGLUT3 subunits in the synaptoneurosomes of the NAc. n = 9, p < 0.05. Data were normalized to values in the sham group. Error bars show mean and s.e.m.

To examine the expression patterns of different VGLUTs in the NAc in the chronic pain state, we used Western blot analysis to measure the protein levels of VGLUT1-3 from the NAc in SNI- or sham-treated rats. We isolated synaptoneurosome fractions from the NAc in these rats 14 days after surgery. Synaptoneurosome fractions contain both pre- and postsynaptic terminals. As over 90% of neurons in the NAc are MSNs, these fractions likely reflect synaptic terminals at MSN synapses. In this case, synaptoneurosome fractions were used to assess the presynaptic expression of VGLUTs. We observed a 30% decrease in the level of VGLUT1 in SNI-treated animals (Figure 2A, n=14-15, p < 0.05). Because VGLUT1 is predominantly expressed in cortical structures such as cerebral cortex, hippocampus, and cerebellar nuclei, our data indicate that chronic pain causes a decrease in the glutamate release from these regions. The level of VGLUT2, however, remained unchanged (Figure 2B, n=15, p > 0.05), suggesting that summed glutamate inputs from thalamus and brainstem to the NAc, mainly mediated by VGLUT2, are likely unaltered in the pain state at a gross biochemical level. Finally, VGLUT3 levels also showed a 20% decrease in chronic pain animals (Figure 2C, n=9, p < 0.05), suggesting a decreased capacity for glutamate co-release onto the MSNs in the NAc from non-glutamatergic neurons.

### Repeated sucrose ingestion increases VGLUT 3 expression in the NAc

**Figure 3:**
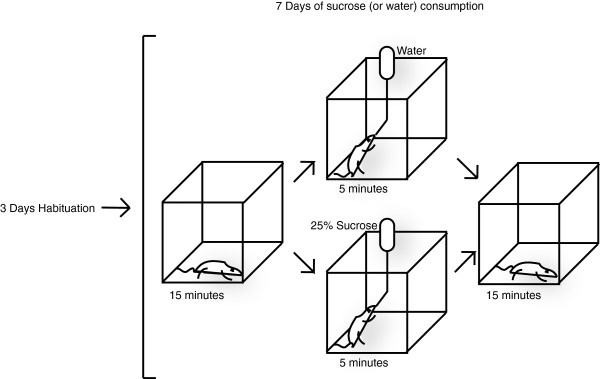
**Sucrose Ingestion Paradigm.** Adult male rats were transported to a test room for 3 consecutive days. The rats were then placed in a chamber for the next 7 days, exposed to bottles containing either 25% sucrose or water (control) for five min on each of these days.

Next we examined VGLUT expressions in the presence of chronic consumption of sucrose, a natural reward. We recently developed a sucrose ingestion paradigm to model the effects of a natural, orosensory reward (Figure 3) [7]. Here, adult male rats were transported to a test room on three consecutive days to acclimate to the test environment. Subsequently, the rats were given 5 minutes access to 25% sucrose solution for 7 consecutive days. During each of these conditioning days, rats were exposed to bottles containing either 25% sucrose solution or water (control) for 5 min. This brief, non-contingent access to a highly palatable (sucrose) solution allowed us to investigate the cumulative effect of repeated consumption of a natural reward (Figure 3). We recently reported that this paradigm results in psychomotor hyperactivity, a behavioral trait indicative of reward seeking, as well as synaptic modifications in the NAc [7].

**Figure 4:**
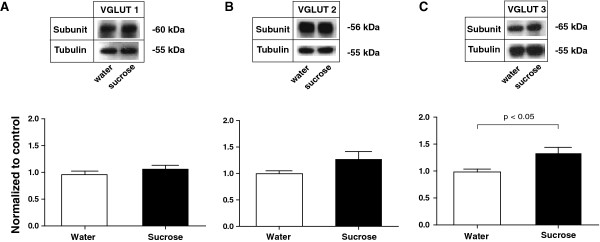
**Repeated Sucrose Ingestion Causes a Selective Increase in VGLUT3 Expression in the NAc. (A)** Repeated ingestion of sucrose caused no changes in VGLUT1 subunits in the synaptoneurosomes of NAc. Student’s t test, n = 8-9, p > 0.05. **(B)** Repeated ingestion of sucrose causes an increased trend for the level of VGLUT2 subunits in the synaptoneurosomes of the NAc. n =11, p > 0.05. **(C)** Repeated ingestion of sucrose caused an increase in VGLUT3 subunits in the synaptoneurosomes of the NAc. n =10, p < 0.05. Data were normalized to values in the control group. Error bars show mean and s.e.m.

Using this sucrose ingestion model, we isolated the synaptoneurosome fractions of rats in the sucrose group as well as rats in the water (control) group. We found that after 7 days of sucrose consumption, there was minimal difference in the synaptoneurosomal expression of VGLUT1 levels in the sucrose group and the control group (Figure 4A, n = 8-9, p > 0.05). This is in contrast to our finding of decreased VGLUT1 expression in rats that experienced chronic pain. Meanwhile, we found that there was an increased trend of VGLUT2 in the sucrose group compared with control group (Figure 4B, n = 11). However, this increase was not statistically significant (p > 0.05). Interestingly, we observed a significant increase in the level of VGLUT3 (~40%) in the sucrose group (Figure 4C, n =10, p < 0.05). These data suggest that an increase in the transport of glutamate to the NAc from primarily non-glutamatergic neurons may occur with natural rewards. This is the opposite of what we found in the chronic pain model, where a decrease of VGLUT3 was observed in SNI-treated rats.

## Discussion

In this study, we examine how natural rewards and pain alter the expression of VGLUTs in the NAc. We find that levels of VGLUT1 and VGLUT3 are decreased with chronic pain, and that VGLUT3 level is increased with chronic administration of natural rewards. These results provide an important step in the understanding of reward circuitry resulting from induction of appetitive and aversive states.

Here we use a behavioral protocol that mimics repeated natural reward consumption [7]. In a previous report, we used this model to show that sucrose consumption increases psychomotor hyperactivity. Psychomotor hyperactivity has been used as an index for addiction-like behaviors, and thus our model provides a predictable behavioral response to chronic consumption of a natural reward. In addition, using this model we have previously shown that chronic sucrose consumption leads to the formation of CPARs at the postsynaptic sites of MSNs. In our current study, we use this same behavioral paradigm for natural rewards to assess presynaptic changes in the NAc [7]. At the same time, we compare these presynaptic changes occurring in response to natural rewards with changes that occur in a chronic pain state, a behavioral condition with contrasting hedonic value.

VGLUT1 is primarily expressed in cortical structures, and it has been shown that the NAc receives gluta-matergic inputs from the PFC and hippocampus in particular [3]. Interplay between hippocampal and PFC glutamate inputs is thought to provide synaptic plasticity in the MSNs to regulate reward learning [39,40]. Our finding that VGLUT1 level is decreased by chronic pain indicates that there may be a diminution of these afferent inputs. This decrease can be the result of decreased expression, altered connectivity, or loss of afferent fibers. Previous reports of grey matter loss in the PFC in chronic pain states suggest the latter possibility [9,41]. On the other hand, the level of VGLUT1 is unaltered in the chronic sucrose administration model. Cortical inputs are well known to regulate reward seeking behaviors [8]. A lack of change in VGLUT1 levels in our study on natural rewards suggests that this top down control may be preserved in the presence of natural rewards.

In contrast to VGLUT1, VGLUT2 is primarily expressed in subcortical structures [20,21]. Whereas the brainstem provides a minor afferent input to the NAc, the thalamus is a major source of glutamate for the NAc [42,43]. In our study, there is an increased trend for VGLUT2 with repeated sucrose consumption, but this trend is not statistically significant. In our model of natural reward, sucrose likely stimulates the orosensory circuit, and the thalamus is a key locus in this circuit. The thalamo-striatal circuit is also known to play an important role in reinstatement of reward learning [44], and thalamic projections have been shown to modify dopaminergic inputs to the NAc in the presence of natural rewards. At the same time, however, VGLUT2 has also been found in dopaminergic neurons from the VTA that project to the NAc [26-29]. Its presence in these neurons has been proposed to allow the release of glutamate from VTA dopamine neurons [45]. This synergy between glutamate and dopamine signaling may be critical for the plasticity of postsynaptic AMPA receptors [46]. In our study, VGLUT2 appears to be increased in the NAc with repeated sucrose ingestion. This increased trend, while not statistically significant, nevertheless suggests that orosensory rewards may either strengthen thalamo-striatal connectivity and/or promote the co-release of glutamate and dopamine. In contrast, VGLUT2 levels are unaltered in the pain state.

Whereas VGLUT1 and 2 are expressed in typical glutamatergic neurons, VGLUT3 is often expressed on serotonergic, GABAergic, or cholinergic neurons and may regulate the co-release of glutamate and serotonin, GABA, or acetylcholine from presynaptic terminals [32]. In the striatum, VGLUT3 is highly expressed in the cholinergic interneurons [33,34]. Although these interneurons comprise of a minority of the neuronal population in the NAc, their inputs to the MSN have been shown to regulate food satiety and addiction to drugs [47-49]. We find here that in the NAc, VGLUT3 is increased in rats that consume sucrose repeatedly, and decreased in rats that have chronic pain. In the NAc, VGLUT3 is involved in the co-release of glutamate from these cholinergic neurons [33,34]. Thus, there are two possible interpretations of our data. First, the change in VGLUT3 levels may represent only a change in the extent of co-release of glutamate, without a concurrent change in acetylcholine release. In this scenario, there may be a de-coupling of glutamate and acetylcholine release from these interneurons. This may serve as a mechanism to selectively control glutamate outputs locally from these neurons without compromising their cholinergic outputs. Cholinergic outputs from these interneurons have been shown to modulate the excitability of MSNs and regulate their responsiveness to additional dopaminergic and glutamatergic inputs [35-37]. This decoupling mechanism allows the preservation of this cholinergic tone and its subsequent regulation of dopamine and glutamate responsiveness of the MSNs. In this case, pain may only decrease glutamate input in the MSNs locally, but modulatory cholinergic inputs are preserved, allowing continued cholinergic gating of inputs from other neuronal sources. Likewise, natural rewards may selectively increase glutamate inputs to the MSNs, without changes in the cholinergic tone in the NAc. A second possible interpretation of our VGLUT3 data is that there is an overall alteration in the presynaptic inputs from these cholinergic interneurons, and both acetylcholine and glutamate release are simultaneously modified by pain or natural reward. In this scenario, the overall neuronal activities of these cholinergic neurons can be modified by the hedonic state of the animal. For example, in the pain state, in addition to a decrease in excitatory glutamate inputs, a reduction of cholinergic inputs may also occur that can alter the responsiveness of the MSNs to dopamine and glutamate inputs from other brain regions as well. In either case, innervation from cholinergic interneurons responds to the hedonic value, where natural rewards increase the capacity for glutamate release, and pain decreases it. Finally, VGLUT3 is also expressed in serotonergic neurons in the raphe nuclei and a subset of GABAergic interneurons in the cortex and hippocampus [20,30,31]. Thus, it is also possible that alterations in VGLUT3 levels observed in our study correspond to changes in inputs from these more distant neuronal populations. Regardless of the exact source of VGLUT3, this opposing expression pattern in pain vs. reward state signals a novel form of synaptic control in the NAc.

## Conclusion

We find that chronic consumption of sucrose, a natural reward, increases VGLUT3 expression in the NAc. Chro-nic pain, however, decreases VGLUT1 and VGLUT3 levels. These results suggest that natural rewards and pain have distinct but important effects on the glutamatergic inputs to the NAc. This study provides the foundation for a better understanding of reward circuitry in rewarding and painful states.

## Methods

### Animals

All procedures in this study were approved by the New York University School of Medicine Institutional Animal Care and Use Committee (IACUC) as consistent with the National Institute of Health (NIH) *Guide for the Care and Use of Laboratory Animals* (publication number 85–23) to ensure minimal animal use and discomfort. Male Sprague–Dawley rats were purchased from Taconic Farms, Albany, NY and kept in the NYU Langone Medical Center’s Central Animal Facility, with controlled humidity, room temperature, and 12-h (6:30 AM to 6:30 PM) light–dark cycle. Food and water were available *ad libitum*. Animals arrived to the animal facility at 300 to 350 grams and were given on average 10 days to adjust to the new environment prior to the onset of any experiments.

### Spared nerve injury (SNI) surgery

The Spared Nerve Injury (SNI) surgery was previously described in detail [38]. Briefly, under Isoflurane anesthesia (1.5 to 2%), the skin on the lateral surface of the right thigh of the rat was incised and a section made through the biceps femoris muscle to expose three branches of the sciatic nerve: sural, common peroneal and tibial nerves. The common peroneal and tibial nerves were tied with non-absorbent 5.0 silk sutures at the point of trifurcation. The nerves were then cut distal to the knot, and about 3 to 5 mm of the distal ends were removed. In sham surgeries (control), above nerves were dissected but not cut. Muscle layer was then sutured closed with absorbent 4.0 antibacterial sutures, while skin was stapled. Staples were removed prior to behavioral testing. Animals were sacrificed 14 days after SNI or sham surgery.

### Sucrose self-administration paradigm

The sucrose self-administration procedure was performed as previously reported. Rats were transported to the test room 3 consecutive days for 2 h/day in their home cages. On the fourth day, after 15-min in the chamber, a bottle with a bead stopper was introduced through the top of the chamber and stabilized. This bottle was introduced for 5 min, and rats were allowed to drink *ad libitum*, and each rat consumed at least 1 g of sucrose by the third session. The bottle was removed from the top of the chamber after 5-min, and the rats remained in the chamber for an additional 15-min after bottle removal. This procedure was repeated identically for 7 consecutive days. On the day of sacrifice, rats were decapitated by guillotine, and tissue samples were collected on ice.

### Subcellular fractionation and Western blotting

Rats were deeply anesthetized with isoflurane (5%) and decapitated immediately. Brains were quickly removed and nucleus accumbens were collected on ice. Whole cell and synaptosome fractions were prepared as described previously [7]. To prepare synaptoneurosome fractions, nucleus accumbens samples were homogenized in an ice-cold solution A (0.32 M sucrose, 1 mM NaHCO_3_, 1 mM MgCl_2_, 0.5 mM CaCl_2_, 0.1 mM PMSF and 1× Complete Protease Inhibitors (Roche Applied Science). Homogenates were centrifuged at 4,000 rpm for 10 min. The supernatant was collected and the pellet re-homogenized in solution A and centrifuged again at 3,000 rpm for 10 min. Combined supernatants were subjected to a second centrifugation at 3,000 rpm for 10 min. Supernatants were then spun at 14,000 rpm for 30 min. Pellet was resuspended in solution B (0.32 M sucrose, 1 mM NaHCO_3_) and homogenized. Homogenate was layered on top of a 5 mL 1 M sucrose and 1.2 M sucrose gradient and centrifuged at 30,000 rpm for 2 hours. Purified synaptosomes were collected at the 1 M and 1.2 M sucrose interface, suspended in solution B and centrifuged at 40,000 rpm for 45 min. Synaptosomal pellets were resuspended in 25 mM TRIS with 4% SDS. Equal amounts of fractions (5–10 μg) were loaded on SDS-PAGE gels and analyzed by Western blot as previously described. Fractions were analyzed by Western blot on SDS-PAGE gels as described previously [48]. The following antibodies were used: VGLUT1 (1:1,000, Millipore), VGLUT2 (1:1,000, Millipore), VGLUT3 (1:500, Millipore) and tubulin (1:30,000, Sigma).

### Animal behavioral tests

#### Mechanical hypersensitivity test

A traditional Dixon up-down method with von Frey filaments was used to measure mechanical hypersensitivity as described previously [38]. In brief, rats were individually placed into plexiglass chambers over a mesh table and acclimated for 20 min before the onset of examination. Beginning with 2.55 g, von Frey filaments in a set with logarithmically incremental stiffness (0.45, 0.75, 1.20, 2.55, 4.40, 6.10, 10.50, 15.10 g) were applied to the lateral 1/3 of right paws (in the distribution of the sural nerve) of rats. 50% withdrawal threshold was calculated as described previously [38].

#### Cold hypersensitivity test

Animals were individually placed into plexiglass chambers as above and acclimated for 20 min. A drop of acetone was applied to the lateral plantar surface of the paws (in the distribution of the sural nerve). As previously described [38], the following scoring system was applied. 0: no visible response or startle response lasting < 0.5 second; 1: paw withdrawal lasting < 5 seconds; 2: withdrawal lasting 5 to 10 seconds, +/− licking of the paws; 3: prolonged repetitive withdrawal lasting > 10s. Acetone was applied 5 times to each paw, and an average score was calculated. Cold hypersensitivity tests were typically done after mechanical allodynia tests on the same day, and observers were blinded to the test conditions.

### Data analysis and statistics

For Western blots, unpaired two-tailed Student’s t tests were used to analyze the proteins levels in SNI- versus sham-treated rats and to analyze water vs. sucrose consuming rats. The results of behavioral experiments were given as mean ± SEM. For mechanical hypersensitivity, a two-way ANOVA with repeated measures, followed by *post hoc* multiple pair-wise comparison Bonferroni tests was used to compare the 50% withdrawal threshold of SNI- and sham-treated rats. Cold hypersensitivity was analyzed using a two-way ANOVA with repeated measures, followed by *post hoc* multiple pair-wise comparison Bonferroni tests. For all tests, a *p* value < 0.05 was considered statistically significant. All data were analyzed using GraphPad Prism Version 5 software (GraphPad, La Jolla, CA).

## Competing interests

The authors declare that they have no competing financial interests.

## Authors’ contributions

DST, EBZ and JW conceived and designed the study. DT, ML, DX, SEE, YG, and TM performed the experiments. DT and ML contributed equally to this manuscript. All authors read and approved the final manuscript.
